# Transcriptional profiling of immune responses in NHPs after low-dose, VSV-based vaccination against Marburg virus

**DOI:** 10.1080/22221751.2023.2252513

**Published:** 2023-09-12

**Authors:** Cecilia A. Prator, Brianna M. Dorratt, Kyle L. O’Donnell, Justin Lack, Amanda N. Pinski, Stacy Ricklefs, Craig A. Martens, Ilhem Messaoudi, Andrea Marzi

**Affiliations:** a Laboratory of Virology, Division of Intramural Research, National Institute of Allergy and Infectious Diseases, National Institutes of Health, Hamilton, MT, USA; b Department of Microbiology, Immunology, and Molecular Genetics, College of Medicine, University of Kentucky, Lexington, KY, USA; c NIAID Collaborative Bioinformatics Resource (NCBR), National Institutes of Allergy and Infectious Diseases, National Institutes of Health, Bethesda, MD, USA; d Department of Molecular Microbiology, Washington University School of Medicine, St. Louis, MO, USA; e Research Technology Branch, Division of Intramural Research, National Institutes of Allergy and Infectious Diseases, National Institutes of Health, Rocky Mountain Laboratories, Hamilton, MT, USA

**Keywords:** Filovirus, Marburg virus Angola, MARV, vesicular stomatitis virus, nonhuman primate, host response

## Abstract

Infection with Marburg virus (MARV), the causative agent of Marburg virus disease (MVD), results in haemorrhagic disease and high case fatality rates (>40%) in humans. Despite its public health relevance, there are no licensed vaccines or therapeutics to prevent or treat MVD. A vesicular stomatitis virus (VSV)-based vaccine expressing the MARV glycoprotein (VSV-MARV) is currently in clinical development. Previously, a single 10 million PFU dose of VSV-MARV administered 1–5 weeks before lethal MARV challenge conferred uniform protection in nonhuman primates (NHPs), demonstrating fast-acting potential. Additionally, our group recently demonstrated that even a low dose VSV-MARV (1000 PFU) protected NHPs when given 7 days before MARV challenge. In this study, we longitudinally profiled the transcriptional responses of NHPs vaccinated with this low dose of VSV-MARV either 14 or 7 days before lethal MARV challenge. NHPs vaccinated 14 days before challenge presented with transcriptional changes consistent with an antiviral response before challenge. Limited gene expression changes were observed in the group vaccinated 7 days before challenge. After challenge, genes related to lymphocyte-mediated immunity were only observed in the group vaccinated 14 days before challenge, indicating that the length of time between vaccination and challenge influenced gene expression. Our results indicate that a low dose VSV-MARV elicits distinct immune responses that correlate with protection against MVD. A low dose of VSV-MARV should be evaluated in clinical rails as it may be an option to deliver beneficial public health outcomes to more people in the event of future outbreaks.

## Introduction

Infection with Marburg virus (MARV), a member of the *Filoviridae* family, results in haemorrhagic disease with high case fatality rates in humans [1,2]. Since its discovery in 1967, MARV has caused over a dozen documented sporadic outbreaks in Africa, with the most recent currently ongoing in Equatorial Guinea and the United Republic of Tanzania [3–7]. Despite its potential threat to local and global public health, there are no licensed vaccines or therapeutics that can be deployed in the event of an MVD outbreak [8,9].

Currently, several vaccine candidates are in various stages of preclinical development and clinical trials, including a vesicular stomatitis virus (VSV)-based vaccine expressing the MARV-Angola glycoprotein (VSV-MARV-GP) [9,10]. A single dose of 10 million plaque-forming units (PFU) of VSV-MARV administered 5 weeks before lethal MARV challenge conferred uniform protection in nonhuman primates (NHPs) [10]. Further inquiry demonstrated the fast-acting potential of VSV-MARV, as NHPs were protected from disease and fatality as early as 7 days after vaccination [11]. We also investigated the relationship between vaccine dose and protection since establishing the minimum protective dose will be essential for delivering the maximum beneficial public health outcomes during an outbreak. Our group recently demonstrated that even a low dose (1000 PFU) of VSV-MARV protected NHPs 7 days before virus challenge [12].

Despite its efficacy in NHPs, the mechanism through which VSV-MARV confers protection at both a low dose and rapid time frame still requires further investigation for clinical application. Transcriptional profiling (e.g. RNA-sequencing) is a powerful approach to detect gene expression changes and transcriptional signatures [13] induced by VSV-MARV vaccination and subsequent MARV challenge. A previous study showed that NHPs vaccinated both 7 and 14 days before challenge with a single dose of 10 million PFU VSV-MARV were protected against MVD [11]. Transcriptional analysis revealed that vaccination with VSV-MARV elicited an antiviral response. After MARV challenge, only a very small number of transcriptional changes were observed apart from genes associated with antiviral response observed in both vaccination groups. Another group of NHPs was vaccinated 7 days before challenge with a similar VSV-based Ebola virus (EBOV) vaccine, VSV-EBOV, and served as control group. These NHPs exhibited a robust inflammatory response associated with the hallmark cytokine storm observed after lethal challenge with filoviruses, before succumbing to MVD [11].

In this study, we investigated longitudinal transcriptional changes in the whole blood of NHPs vaccinated with a low dose of VSV-MARV (1000 PFU) either 14 (d-14) or 7 (d-7) days before lethal MARV challenge [12].

## Materials and methods

### Ethics statement

All the work involving infectious MARV was performed following standard operating procedures approved by the Rocky Mountain Laboratories (RML) Institutional Biosafety Committee in the maximum containment laboratory at the RML, Division of Intramural Research, National Institute of Allergy and Infectious Diseases, National Institutes of Health. NHP work was performed in strict accordance with the recommendations described in the Guide for the Care and Use of Laboratory Animals of the National Institute of Health, the Office of Animal Welfare, and the United States Department of Agriculture and was approved by the RML Animal Care and Use Committee (ACUC). NHPs were housed in adjoining individual primate cages that enabled social interactions, under controlled conditions of humidity, temperature, and light (12-h light:12-h dark cycles). Food and water were available *ad libitum*. NHPs were monitored and fed commercial monkey chow, treats, and fruit at least twice a day by trained personnel. Environmental enrichment consisted of commercial toys, music, and video. Procedures were conducted by trained personnel on anaesthetized NHPs. All efforts were made to ameliorate NHP welfare and minimize suffering per the Weatherall Report on the use of NHPs in research (https://royalsociety.org/policy/publications/2006/weatherall-report/). Endpoint criteria based on clinical score parameters as specified and approved by the RML ACUC were used to determine when animals were humanely euthanized.

### NHP study design

Whole blood samples from six male and six female cynomolgus macaques (*Macaca fascicularis*) 3–4 years of age and 2.1–4.5 kg in weight were collected during a previous study [12]. One group of cynomolgus macaques (*n* = 4; 2 male and 2 female) were vaccinated with a single IM injection of 1000 PFU VSV-MARV and challenged 14 days post-vaccination. Clinical exams including a blood draw were conducted on −14, −11, −7, −4, 0, 1, 3, 6, 9, 14, 21, 28, 35, and 42 days post-challenge (DPC). Two additional groups of cynomolgus macaques (*n* = 4; each 2 male and 2 female) were IM-vaccinated with 1000 PFU VSV-MARV or VSV-EBOV and challenged 7 DPV. Clinical exams including a blood draw were conducted on −7, −4, 0, 1, 3, 6, 9, 14, 21, 28, 35 and 42 DPC. An IM injection of 1000 PFU MARV-Angola (confirmed by back-titration) as previously described [10] served as lethal challenge for all NHPs. The animals were observed at least twice daily for clinical signs of disease according to a RML ACUC-approved scoring sheet and humanely euthanized when they reached endpoint criteria. The study ended 42 DPC when all surviving animals were humanely euthanized.

### cDNA library preparation and RNA-sequencing

The study design and summary of NHP whole blood samples used for RNA-sequencing are summarized in Table S1. Quality and concentration of RNA were verified on an Agilent 2100 Bioanalyzer and 100 ng RNA was used for library preparation. Ribosomal RNA was depleted using the NEBNext rRNA depletion kit following manufacturer’s instructions. The remaining RNA was fragmented at 94°C for 7 minutes. Libraries were prepared using the Illumina® Stranded Total RNA Prep kit and 16 cycles of PCR amplification were used for index addition and library fragment enrichment. A total of 80 RNA-Seq samples were pooled and sequenced on NovaSeq 6000 S4 using paired-end sequencing.

### Bioinformatic analysis

In each group, all available RNA samples after vaccination and challenge were compared to time of vaccination (baseline). For consistency, we modelled this analysis after previous transcriptomic analyses of samples from VSV-MARV-vaccinated and MARV-challenged NHPs using the RNA-Seq workflow from the systemPipeR package [10,11,14]. The Trim Galore package was used to trim raw sequences to 70 bp and an average Phred score of 30. Trimmed sequences were aligned to the Macaca fascicularis genome “Macaca_fascicularis.Macaca_fascicularis_6.0.dna.toplevel.fa” using hisat. Genes were annotated with Ensembl file “Macaca_fascicularis.Macaca_fascicularis_6.0.106.gtf.” Raw gene counts were identified by the summarizeOverlaps package before being converted to transcripts per kilobase million (TPM).

Gene expression analysis was performed using three approaches: (1) EdgeR, (2) STEM, and (3) MaSigPro [15–17]. The EdgeR package identifies differentially expressed genes (DEGs) in one condition relative to another. DEGs were then filtered for only those with an FDR ≤ 0.05, fold change ≤ −1 or ≥ 1, and encoded for human protein-coding homologues. The Short Time Series Expression Miner (STEM) software was used to identify significant patterns of longitudinal gene expression change [15] within each condition. The MaSigPro package was used to identify significant longitudinal gene expression profile changes between conditions using a two-way forward regression strategy. Functional enrichment of DEGs was performed with Metascape [http://metascape.org] [18]. Transcripts specific to *Marburg marburgvirus* were quantified using Kraken2 [19] and the standard database containing all bacterial and viral sequences available in RefSeq as of December 9, 2022 (https://benlangmead.github.io/aws-indexes/k2). All heatmaps, bar plots, bubble plots, and Venn diagrams were made using R package ggplot2.

## Results

### VSV-MARV vaccination induces transcriptional changes consistent with an antiviral response in NHPs vaccinated 14 or 7 days before challenge

**Figure 1.**
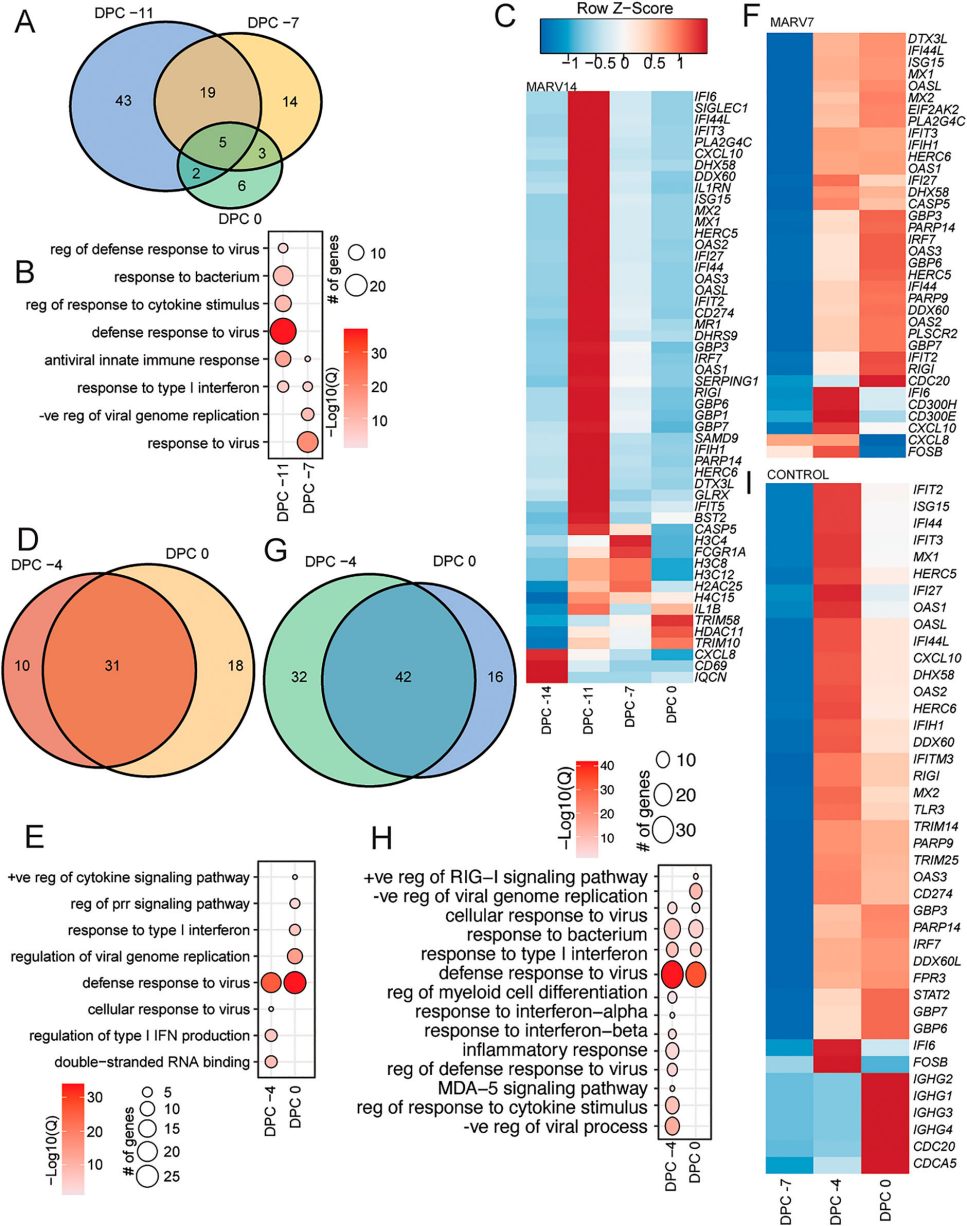
VSV-MARV vaccination induces transcriptional changes. (A) Venn diagram of DEGs detected at DPC −11, −7, and 0 for the MARV14 group relative to baseline (DPC −14). (B) Functional enrichment of DEGs in panel A. (C) Heatmap of DEGs mapping to GO terms from panel B. (D) Venn diagram of DEGs detected at DPC −4 and 0 for the MARV7 group relative to baseline (DPC −7). (E) Functional enrichment of DEGs in panel D. (F) Heatmap of DEGs mapping to GO terms from panel E. (G) Venn diagram of DEGs detected at DPC −4 and 0 for the control group relative to baseline (DPC −7). (H) Functional enrichment of DEGs in panel G. (I) Heatmap of DEGs mapping to GO terms from panel H. (B, E, H) Size of the bubble indicates the number of genes mapping to the GO term and colour indicates the −log10(Q-value). (C, F, I) Colour indicates *z*-score normalized across each row.

We first examined gene expression changes in response to VSV-MARV vaccination (Table 1). Timepoints collected at −14 (baseline), −11, −7, −4 and 0 DPC were analysed for the day −14 vaccinated VSV-MARV (MARV14) group. Principal component analysis (PCA) revealed clustering for all MARV14 NHPs at all timepoints (Figure S1A). In this group, 69 and 41 DEGs were detected at 3 and 7 days after vaccination (−11 and −7 DPC) and only 16 DEGs were detected at 0 DPC (Table 1, Figure 1(A)).

**Table 1.** Summary of differentially expressed genes (DEGs) after vaccination and relative to baseline for each indicated comparison.

Group	Comparison	No. DEGs
MARV14	DPC −11 vs DPC −14	69
	DPC −7 vs DPC −14	41
	DPC 0 vs DPC −14	16
MARV7	DPC −4 vs DPC −7	41
	DPC 0 vs DPC −7	49
Control	DPC −4 vs DPC −7	74
	DPC 0 vs DPC −7	56

DPC: days post-challenge.

Timepoints collected at −7 (baseline), −4 and 0 DPC were analysed for the day −7 vaccinated VSV-MARV (MARV7) and day −7 vaccinated VSV-EBOV (Control) groups. PCA resulted in clustering for all Control NHPs at all time points (Figure S1B), however, in the MARV7 group, two outliers were identified (Figure S1C). For all analysis these two data points were excluded resulting in clustering shown in Figure S1D. In the MARV7 group, 41 DEGs were detected at −4 DPC and increased to 49 DEGs at 0 DPC (Table 1, Figure 1(D)). For both, the MARV14 and MARV7 groups, functional enrichment analysis demonstrated transcriptional changes indicative of an antiviral response with DEGs enriching to gene ontology (GO) terms “defense response to virus” and “antiviral innate immune response” at 3 days post-vaccination (−11 or −4 DPC, respectively) and “response to virus” at 7 days post-vaccination (−7 or 0 DPC, respectively) (Figure 1(B,C,E,F)).

Gene expression changes were also apparent in the control group at −4 and 0 DPC with 74 and 56 DEGs detected, respectively (Figure 1(G)). In this group, DEGs enriching to GO terms were also associated with an antiviral immune response including “defense response to virus,” “inflammatory response,” and “negative response to viral process” (Figure 1(H,I)).

### Transcriptional profiles differ between MARV7 and MARV 14 groups after challenge

We then analysed gene expression changes in response to MARV challenge in all groups (Table 2). In the MARV14 group, the largest number of DEGs were detected at 3 DPC (127), and then declined to 118, 18, and 18 DEGs at 6, 9, and 14 DPC, respectively. Interestingly, the number of DEGs in the MARV7 group was lower compared to the MARV14 group with 54, 13, 3, and 4 DEGs at 3, 6, 9, and 14 DPC, respectively. In the control group, 204 DEGs were detected at 3 DPC and increased to 899 DEGs 6 DPC during late-stage MVD.

**Table 2.** Summary of differentially expressed genes (DEGs) after challenge and relative to baseline for each indicated comparison.

Group	Comparison	No. DEGs
MARV14	DPC 3 vs DPC −14	127
	DPC 6 vs DPC −14	118
	DPC 9 vs DPC −14	18
	DPC 14 vs DPC −14	18
MARV7	DPC 3 vs DPC −7	54
	DPC 6 vs DPC −7	13
	DPC 9 vs DPC −7	3
	DPC 14 vs DPC −7	4
Control	DPC 3 vs DPC −7	204
	DPC 6 vs DPC −7	899

DPC: days post-challenge.

**Figure 2.**
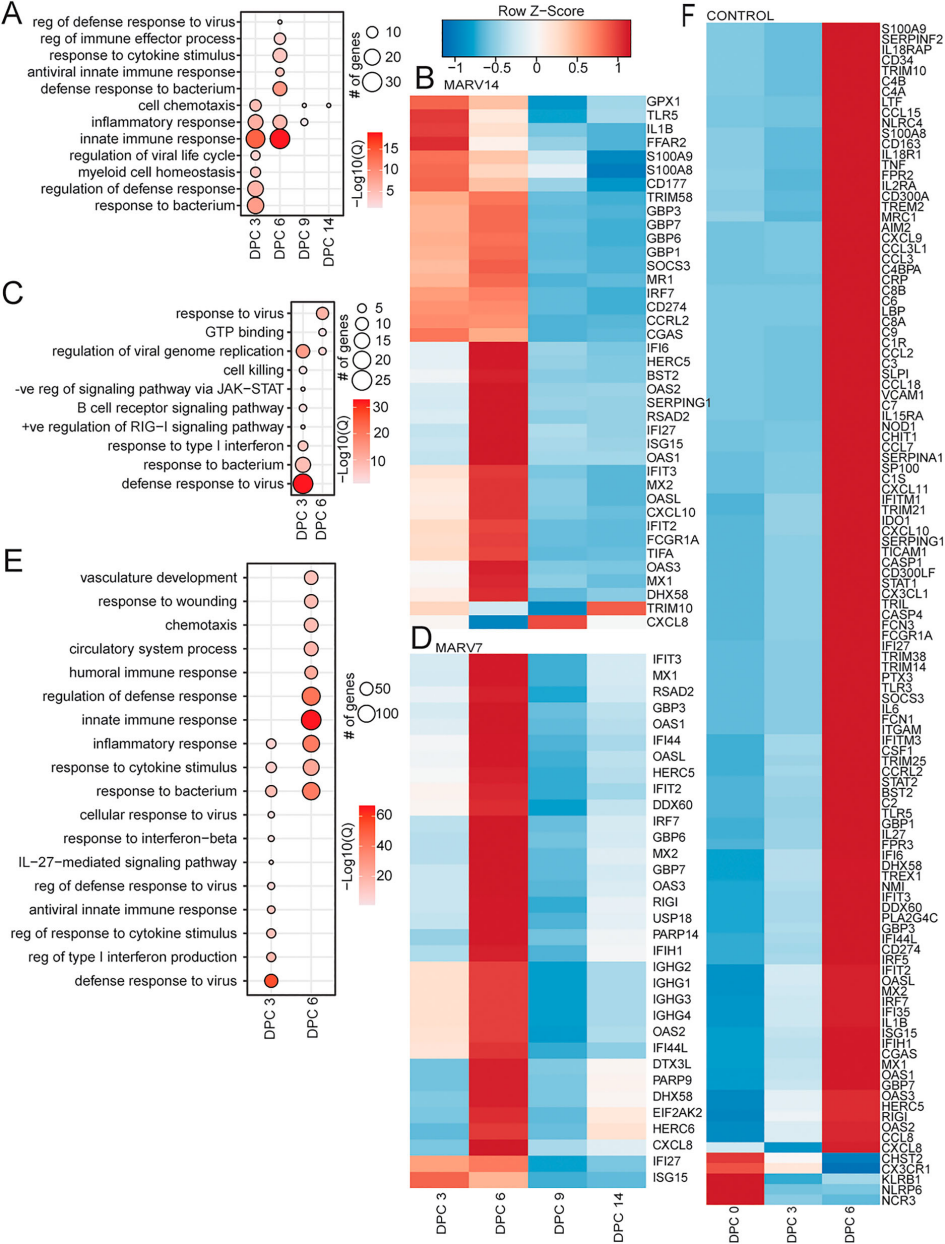
Transcriptional changes in each group after MARV challenge. (A) Functional enrichment of DEGs at DPC 3, 6, 9, and 14 in the MARV14 group relative to baseline (DPC −14). (B) Heatmap of DEGs mapping to GO terms from panel A. (C) Functional enrichment of DEGs at DPC 3, 6, 9, and 14 in the MARV7 group relative to baseline (DPC −7). (D) Heatmap of DEGs mapping to GO terms from panel C. (E) Functional enrichment of DEGs at DPC 0, 3, and 6 in the control group relative to baseline (DPC −7). (F) Heatmap of DEGs mapping to GO terms from panel E. (A, C, E) Size of the bubble indicates the number of genes mapping to the GO term and colour indicates the −log10(Q-value). (B, D, F) Colour indicates *z*-score normalized across each row.

In the MARV14 group, DEGs at 3 and 6 DPC enriched to GO terms “innate immune response,” “inflammatory response,” and “regulation of defense response” (Figure 2(A)). Notable genes in this group included *IL1B*, *S100A8* and *S100A9*, which play a role in the inflammatory response (Figure 2(B)). Genes associated with an antiviral response (*OAS1*, *RSAD2*, *IRF7* and *MX1*) were upregulated early after challenge (3 and/or 6 DPC) but then downregulated at 9 and 14 DPC. In the MARV7 group, DEGs at 3 DPC enriched to GO terms “defense response to virus,” “response to bacterium,” and “regulation of viral genome replication” (Figure 2(C)). In comparison to the MARV14 group less DEGs were upregulated on 3 DPC (Figure 2(A–D)). At 6 DPC DEGs in the MARV 7 group enriched to GO terms “response to virus” and “regulation of viral genome replication.” Similar to the MARV14 group, upregulated DEGs indicative of an antiviral response included *OAS1*, *RSAD2*, *IRF7* and *MX1* (Figure 2(D)).

Functional analysis of the control group revealed a distinct transcriptional profile from both vaccinated groups (Figure 2(E)). At 3 DPC genes enriched similar GP terms compared to the MARV7 and MARV14 groups including to “defense response to virus” (*S100A8*, *S100A9* and *MX2*) and “inflammatory response.” In contrast, at 6 DPC genes enriched to a more diverse set of functional groups related to immune responses including “innate immune response” (*IFIT3*, *OAS3*, *MX1*, *IRF7*, and *STAT2*), “response to wounding,” “vasculature development,” “regulation of defense response,” “circulatory system process,” and “humoral immune response” (Figure 2(E)). This distinct profile was observed on 6 DPC after challenge when NHPs were in the later stages of MVD (Figure 2(F)).

### Inter-group variability before and after virus challenge

**Figure 3.**
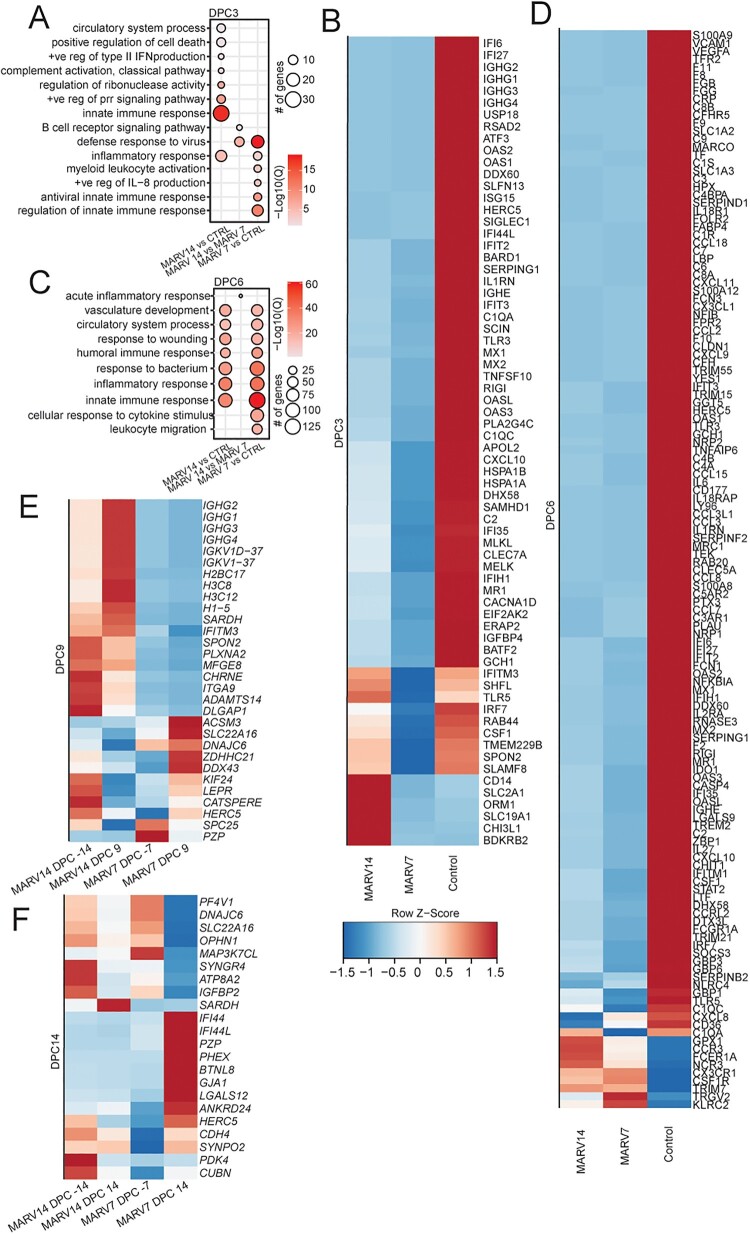
Comparison of transcriptional changes after challenge between groups. (A) Functional enrichment of DEGs between groups at DPC 3. (B) Heatmap of DEGs mapping to GO terms from panel A. (C) Functional enrichment of DEGs between groups at DPC 6. (D) Heatmap of DEGs mapping to GO terms from panel C. (E) Heatmap of DEGs between groups at DPC 9. (F) Heatmap of DEGs between groups at DPC 14. (A, C) Size of the bubble indicates the number of genes mapping to the GO term and colour indicates the −log10(Q-value). (B, D–F) Colour indicates *z*-score normalized across each row.

Gene expression changes between the three groups at each timepoint were compared and are summarized in Table 3. At the time of MARV challenge, there are differences in the number of DEGs between the MARV14 and the other two groups, but interestingly not between the control and MARV7 group. Differences between the MARV14 and MARV7 groups compared to the control group increase at 3 DPC while the differences between the vaccination groups decreases. DEGs are associated with GO terms “innate immune response” (*OAS2*, *OAS3*, and *MX1*), “defense response to virus,” “inflammatory response,” and “regulation of innate immune response” (Figure 3(A,B)). Interestingly, the MARV7 groups present with the lowest number for DEGs at this point (Table 2, Figure 3(B)). By 6 DPC, the DEG differences between the vaccination groups continue to decrease whereas the control group has developed a distinctly different DEG profile (Table 3; Figure 3(C,D)). DEGs are associated with GO terms “innate immune response,” “response to bacterium,” “inflammatory response,” “humoral immune response,” and “response to wounding” (Figure 3(C)). At 9 and 14 DPC, the transcriptomic signature in the MARV7 and MARV 14 groups have majorly returned to baseline with small differences between the groups (Figure 3(E,F)).

**Table 3.** Summary of differentially expressed genes (DEGs) between each group at each time point.

Group	Comparison	No. DEGs
DPC 0	MARV14 vs Control	97
	MARV14 vs MARV7	86
	MARV7 vs Control	0
DPC 3	MARV14 vs Control	114
	MARV14 vs MARV7	58
	MARV7 vs Control	53
DPC 6	MARV14 vs Control	738
	MARV14 vs MARV7	26
	MARV7 vs Control	873
DPC 9	MARV14 vs MARV7	30
DPC 14	MARV14 vs MARV7	22

DPC: days post-challenge.

**Figure 4.**
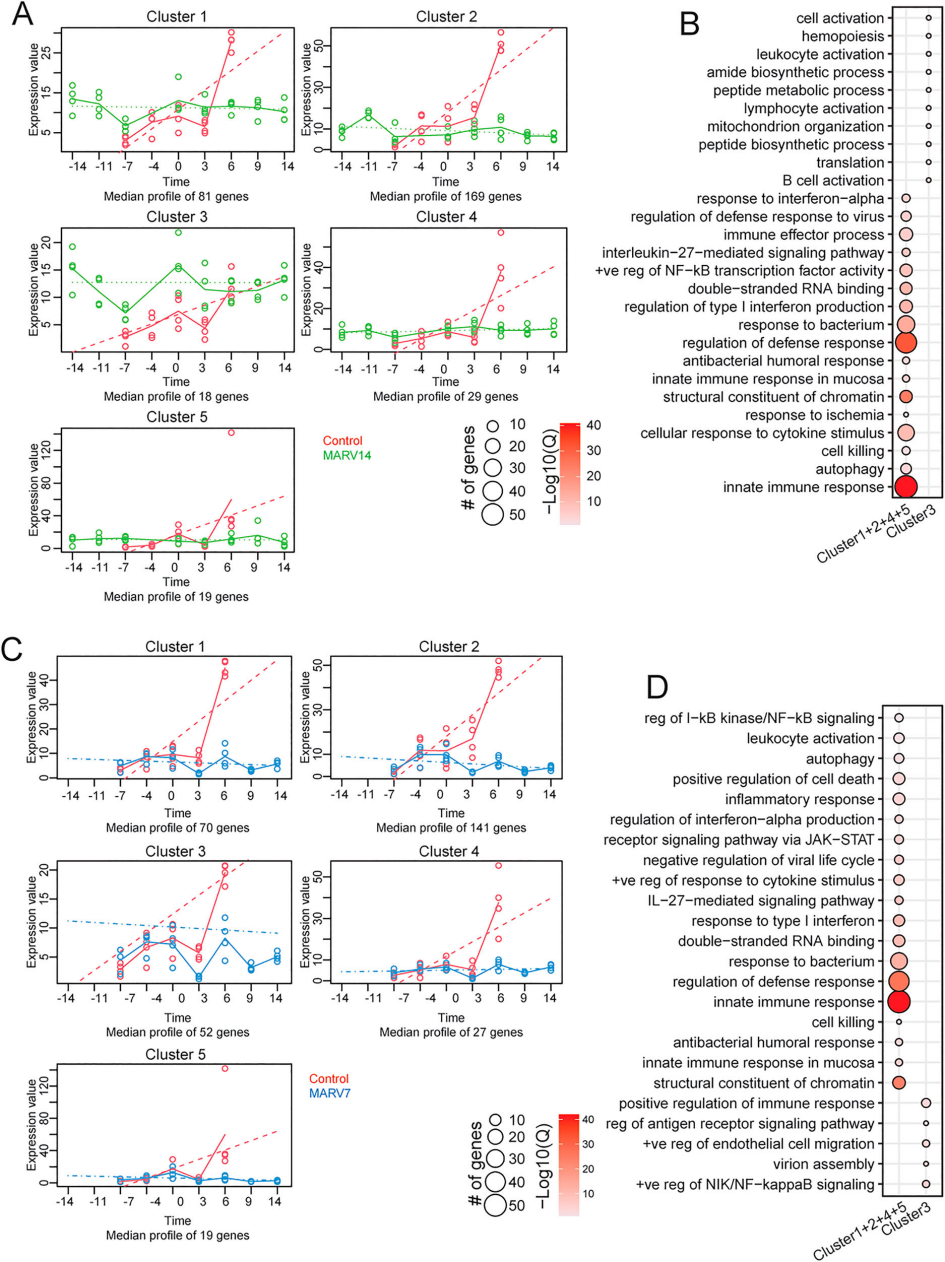
Comparisons of DEGs over the entire study between the MARV14 and MARV7 groups relative to the control group. (A) maSigPro analysis of MARV14 relative to control group identified five gene clusters that have distinct and significant temporal expression differences. (B) Functional enrichment of DEGs within the indicated group from panel A. (C) maSigPro analysis of MARV7 relative to control group identified five gene clusters that have distinct and significant temporal expression differences. (D) Functional enrichment of DEGs within the indicated group from panel C. (B, D) Size of the bubble indicates the number of genes mapping to the GO term and colour indicates the −log10(Q-value).

**Figure 5.**
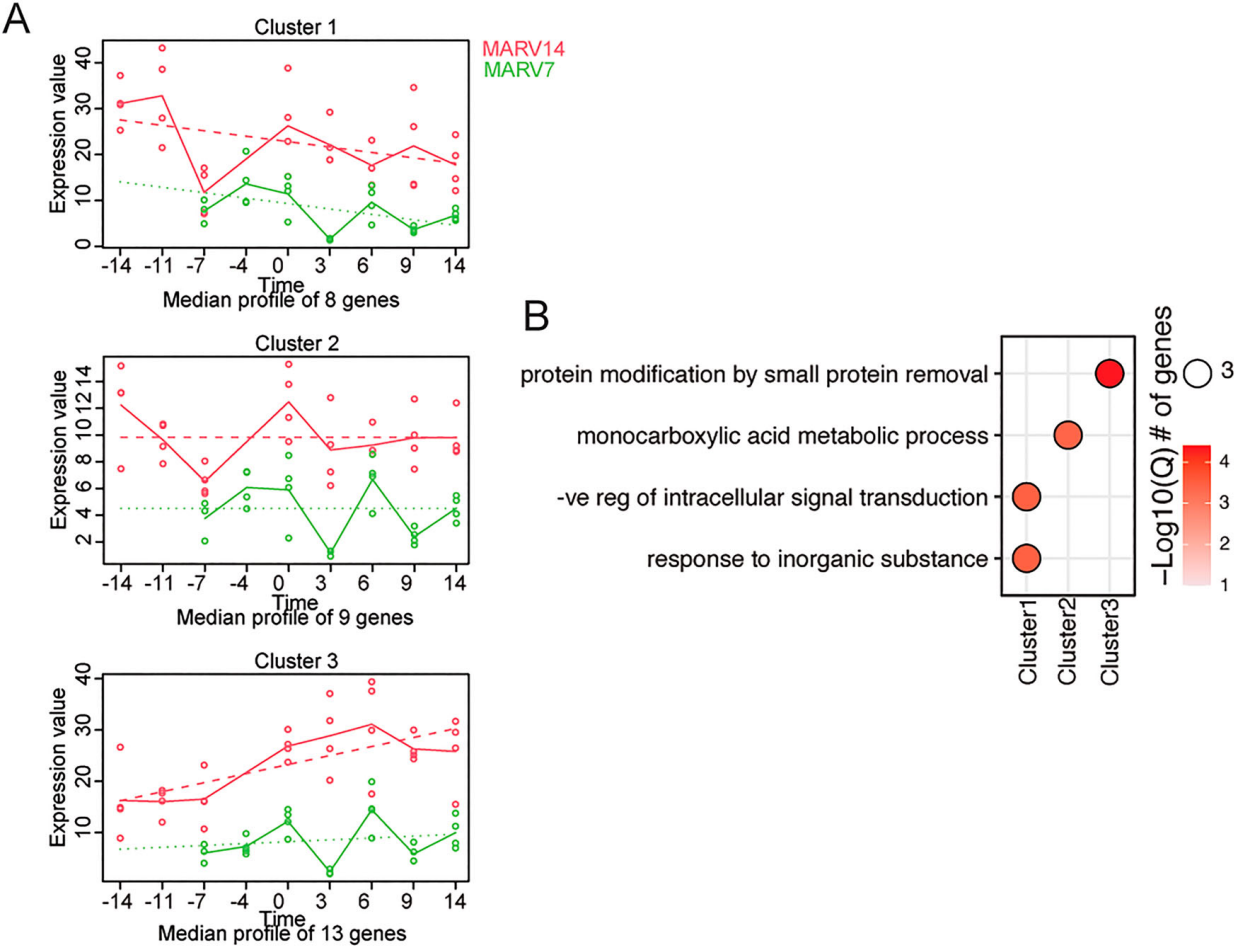
Comparisons of DEGs over the entire study between the MARV14 and MARV7 groups. (A) maSigPro analysis of MARV14 relative to MARV7 group identified three gene clusters that have distinct and significant temporal expression differences. (B) Functional enrichment of DEGs within the indicated group from panel A. Size of the bubble indicates the number of genes mapping to the GO term and colour indicates the −log10(Q-value).

To determine if there were differences in expression over time between the two VSV-MARV vaccination groups and the control group, we used maSigPro (Figure 4). First, we compared either the MARV14 (Figure 4(A)) or the MARV7 group (Figure 4(C)) to the control group to determine if there were significant genes associated with specific temporal patterns that were distinct between each group. We observed two major temporal trends; four clusters in each analysis were genes that showed low expression across the three groups throughout the time-course followed by a peak at the final timepoint in the control group (clusters 1, 2, 4, 5; Figure 4(A)). DEGs that were significantly different applying this analysis between the MARV14 and control groups are involved in “regulation of defense response,” “innate immune response,” “cellular response to cytokine stimulus,” and “immune effector process” (Figure 4(B)). When comparing the MARV7 group to control NHPs, DEGs clustered similarly over time and enriched to similar functional groups including “innate immune response,” “regulation of defense response,” “response to type I interferon,” and “response to bacterium” (Figure 4(D)). Additional analysis compared both vaccinated groups to each other which resulted in five clusters of DEGs that differed between these groups (Figure 5). We identified three clusters documenting a higher number of DEGs over time in the MARV 14 group compared to MARV7 (Figure 5(A)). The MARV7 group exhibited somewhat similar expression patterns in all clusters whereas the MARV14 group had a distinct pattern in cluster 3 (Figure 5(A)). In cluster 1 genes were enriched to “response to inorganic substance” and “negative regulation of intracellular signal transduction.” Cluster 2 showed similar expression pattern with DEGs enriching to “monocarboxylic acid metabolic process.” The genes in cluster 3 enriched to “protein modification by small protein removal.”

## Discussion

Previous work demonstrated that vaccination with a single high dose of VSV-MARV protected NHPs from MVD when administered 7 days before challenge. Our group recently demonstrated that 100% of NHPs were protected after vaccination with a low dose (1000 PFU) of VSV-MARV 7 days before virus challenge [12]. In this study, we used RNAseq to analyse whole blood samples collected after vaccination and challenge from this experiment.

In our pipeline using human homologues for functional analyses, vaccinated NHPs challenged 7 or 14 days after vaccination demonstrated a robust immune defense response early after challenge (3 DPC). We also observed the immediate induction of genes associated with the inflammatory response. This supports previous observations that VSV-MARV primes the immune system to mediate rapid protection against MARV challenge [10,11]. In contrast, a different transcriptional response was observed in control NHPs after MARV challenge. Only a small number of genes associated with “defense response to virus,” “cellular response to virus,” and “antiviral innate immune response” were differentially expressed at 3 DPC. At 6 DPC, we observed a remarkable induction of innate and adaptive immune responses as well as genes associated with interferon and cytokine responses. This explosive immune response corresponds with progression of observable disease in the control NHPs and follows the signature “cytokine storm” described after lethal challenge with MARV [11,12,20].

Previous studies established that VSV-based vaccine protection against filovirus infections is primarily mediated by the humoral response and that there is limited contribution by T cell responses [11,21,22]. Our results indicate that after challenge, T cell activation, differentiation, and lymphocyte-mediated immunity play a role in protection against MVD in the MARV14 group. Additionally, genes enriched to T cell activation and lymphocyte-mediated immunity included CD1C and CD3E which encode proteins that play roles in the T cell receptor complex [23] while PRDX2 is known to contribute to the antiviral activity of CD8 T cells [24]. Genes enriching to T cell activation and differentiation were not enriched in the MARV7 or control groups. These findings are in line with observations made by O’Donnell et al. showing by T cell immune phenotyping assays of the same cohort of NHPs that the low dose of VSV-MARV elicited a more robust CD4+ IFNγ response when compared to NHPs vaccinated with a higher VSV-MARV dose [12]. This implies that a robust T cell response is not necessary for protection but does contribute to protection of NHPs given more time to respond to low dose vaccination. When comparing these results to previously published gene expression patterns generated from NHPs vaccinated with a higher dose of 1 × 10^7^ PFU VSV-MARV, we see a similar pattern. Marzi et al. observed a significant representation of genes enriching to T cell activation after vaccination (11–14 days after vaccination) [11] again confirming that genes associated with T cell mediated immunity may take a longer time to activate after VSV-MARV vaccination. After vaccination, both the higher and lower dose see a smaller initial response (3 days after vaccination) to vaccination, but gene expression changes increase over time to 14 days after vaccination. After challenge, we did not see significant enrichment in the MARV7 vaccinated or control groups. Vaccination with the higher dose also resulted in limited gene expression changes in MARV7 NHPs.

In addition to investigating gene expression changes using day of vaccination samples as a baseline, we conducted time-course analyses comparing gene expression variation over time between all groups. When comparing the MARV14 groups to the control NHPs, genes enriched to “B cell activation” and “antibacterial humoral response” which supports findings made by O’Donnell et al. in humoral response functionality assays on vaccinated NHPs [12]. Genes enriching to “B cell activation” were not evident in the MARV7 group in time-course comparisons, however, genes involved in “regulation of antigen receptor signaling pathway” and “antibacterial humoral response” were upregulated. This further confirms that even at a low dose (1000 PFU), an antigen-specific humoral response drives VSV-based vaccine protection [11,12,22,25]. Another feature unique to vaccinated NHPs, was the expression of genes enriched to functional groups related to “IL-27-mediated signaling pathway.” MARV infects dendritic cells and macrophages early on [26] and secreted cytokines like IL-27 may activate B and T cells to respond to the infection [27–29]. Our results indicate that vaccinated animals were able to better regulate cytokine responses after MARV challenge. Genes enriched to control of the immune response were not observed in the control group who succumbed to disease.

Overall, transcriptional profiling of whole blood samples taken from NHPs vaccinated with a low dose of VSV-MARV revealed gene expression changes indicative of both innate immune responses and T cell activation. The MARV7 group showed limited gene expression changes after challenge when compared to the MARV14 group. Our results indicate that even at a low dose VSV-MARV elicits distinct immune responses that correlate with protection against MVD. Despite increased outbreaks of MARV reported as recently as February 2023 in Equatorial Guinea [30] and March 2023 in the United Republic of Tanzania [31], an approved vaccine to protect vulnerable populations remains unavailable. A low dose of VSV-MARV should be evaluated in clinical trials as it may be an option to deliver beneficial public health outcomes to more people in the event of future outbreaks.

## Supplementary Material

Supplemental Material

## Data Availability

The datasets generated for this study can be found in the following repository: NCBI SRA BioProject, accession number PRJNA918630.
