# Supplementary material for: Transcriptional profiling of immune responses in NHPs after low-dose, VSV-based vaccination against Marburg virus
Source: Emerg Microbes Infect. 2023 Sep 12;12(2):2252513. doi: 10.1080/22221751.2023.2252513 (PMC10498809; doi:10.1080/22221751.2023.2252513)
Supplement: Supplemental Material [file TEMI_A_2252513_SM5437.pdf]

SUPPLEMENTAL MATERIALS

Transcriptional profiling of immune responses in NHPs after low-dose, VSV-based vaccination against Marburg virus

Cecilia A. Prator[1], Brianna M. Doratt[2], Kyle L. O'Donnell[1], Justin Lack[3], Amanda N. Pinski[4], Stacy Ricklefs[5], Craig A. Martens[5], Ilhem Messaoudi[2], and Andrea Marzi[1]

[1] Laboratory of Virology, Division of Intramural Research, National Institute of Allergy and Infectious Diseases, National Institutes of Health, Hamilton, MT, United States
[2] Department of Microbiology, Immunology, and Molecular Genetics, College of Medicine, University of Kentucky, Lexington, KY, United States
[3] NIAID Collaborative Bioinformatics Resource, National Institutes of Allergy and Infectious Diseases, National Institutes of Health, Bethesda, MD, United States
[4] Department of Molecular Microbiology, Washington University School of Medicine, St. Louis, MO, United States
[5] Research Technology Branch, Division of Intramural Research, National Institutes of Allergy and Infectious Diseases, National Institutes of Health, Rocky Mountain Laboratories, Hamilton, MT, United States

Table S1. Study samples.
Figure S1. Principal component analysis of all samples.

	VSV-MARV Day -14 (MARV14)	VSV-MARV Day -7 (MARV7)	VSV-EBOV Day -7 (control)
**DPC -14**	4		
**DPC -11**	4		
**DPC -7**	4	(4) 3	4
**DPC -4**		4	4
**DPC 0**	4	4	4
**DPC 3**	4	(4) 3	4
**DPC 6**	4	4	4
**DPC 9**	4	4	
**DPC 14**	4	4	

Table S1. Study samples. Number of NHP whole blood samples collected at each respective timepoint and used for sequencing and analysis. In the MARV7 group, 2 outliers were identified and removed from the data for all analysis resulting in n=3 for the respective time points.

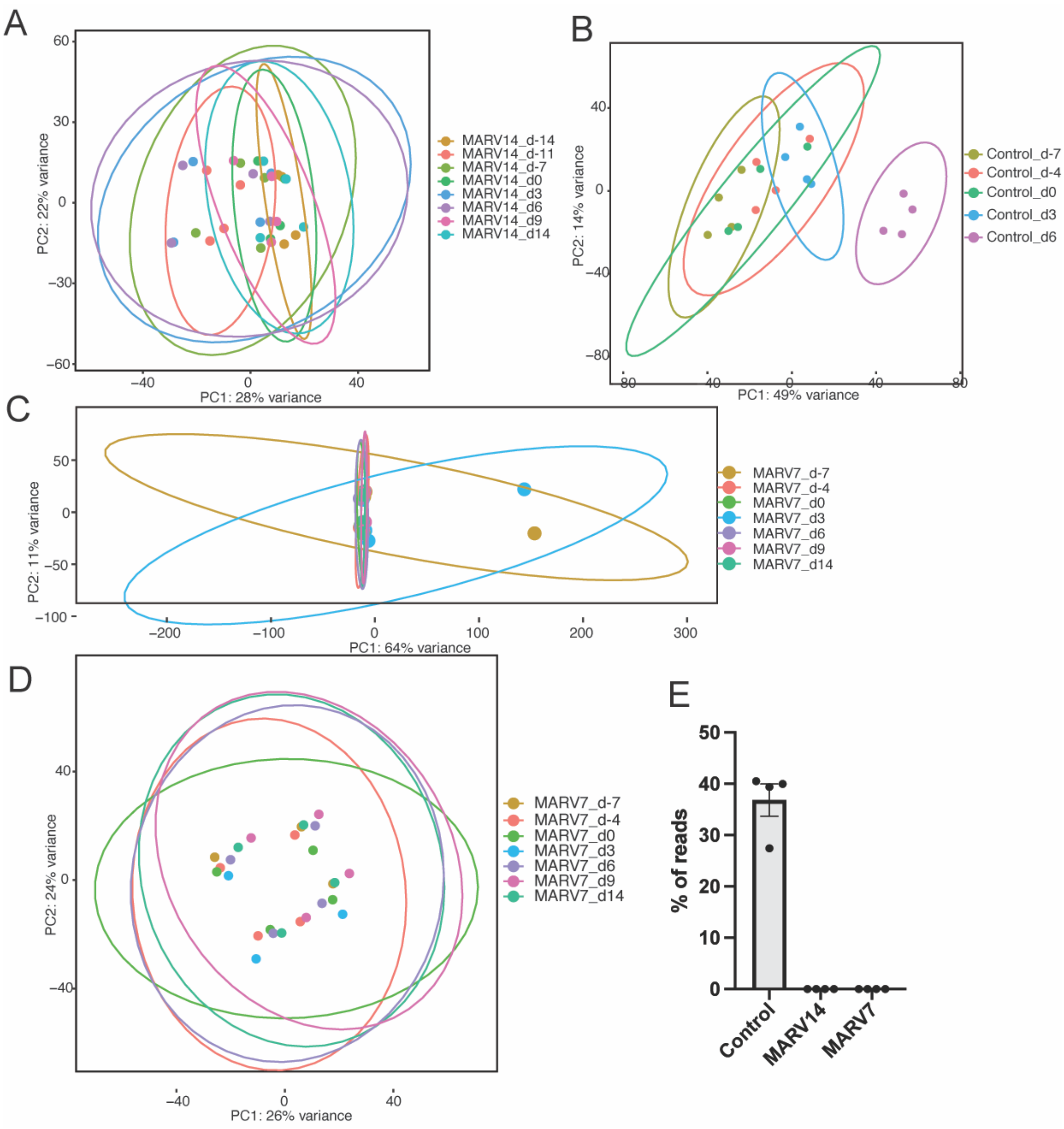

Figure S1. Principal component analysis of all samples. A) Principal component analysis (PCA) of samples from MARV14. B) PCA of samples from the Control group. C) PCA of samples from the MARV7 group identified two outliers confirmed by PcaHubert analysis. Outliers were removed for all downstream analysis. D) PCA of samples from the MARV7 group without outliers. E) Bar plot of the percentage of reads mapping to the MARV genome at 6 DPC. No viral reads were detected at any other timepoint.
